# Data-driven automated control algorithm for floating-zone crystal growth derived by reinforcement learning

**DOI:** 10.1038/s41598-023-34732-5

**Published:** 2023-05-09

**Authors:** Yusuke Tosa, Ryo Omae, Ryohei Matsumoto, Shogo Sumitani, Shunta Harada

**Affiliations:** 1 Anamorphosis Networks, 50 Higashionmaeda-Cho, Nishishichijo, Shimogyo-Ku, Kyoto, 600-8898 Japan; 2 Center for Integrated Research of Future Electronics (CIRFE), Institute of Materials and Systems for Sustainability (IMaSS), Nagoya University, Furo-Cho, Chikusa-Ku, Nagoya, 464-8601 Japan; 3 Department of Materials Process Engineering, Nagoya University, Furo-Cho, Chikusa-Ku, Nagoya, 464-8603 Japan

**Keywords:** Materials science, Theory and computation, Computational methods, Design, synthesis and processing

## Abstract

The complete automation of materials manufacturing with high productivity is a key problem in some materials processing. In floating zone (FZ) crystal growth, which is a manufacturing process for semiconductor wafers such as silicon, an operator adaptively controls the input parameters in accordance with the state of the crystal growth process. Since the operation dynamics of FZ crystal growth are complicated, automation is often difficult, and usually the process is manually controlled. Here we demonstrate automated control of FZ crystal growth by reinforcement learning using the dynamics predicted by Gaussian mixture modeling (GMM) from small numbers of trajectories. Our proposed method of constructing the control model is completely data-driven. Using an emulator program for FZ crystal growth, we show that the control model constructed by our proposed model can more accurately follow the ideal growth trajectory than demonstration trajectories created by human operation. Furthermore, we reveal that policy optimization near the demonstration trajectories realizes accurate control following the ideal trajectory.

## Introduction

The application of informatics has enabled us to realize efficient optimization, automation and advances in materials processing^1–9^. The design of conditions and environments for materials processing has been efficiently optimized using surrogate models built by neural networks or other machine learning algorithms^1,2,6,10–13^. Bayesian optimization can successfully reduce the number of trials for the acquisition of favorable conditions for materials processing^14–17^. On the other hand, some materials processing requires manual control according to information obtained during operation, and is difficult to automate. For example, in floating-zone (FZ) crystal growth, which is used to produce silicon wafers and various kinds of crystalline materials such as semiconductors, oxides, metals, and intermetallic compounds, an operator adaptively controls the input parameters to maintain preferred conditions for single-crystal growth by monitoring the status of the melt in the chamber^18–28^. In the present study, we aimed to construct a control model for automated operation of FZ crystal growth from a small number of operation trajectories.

**Figure 1:**
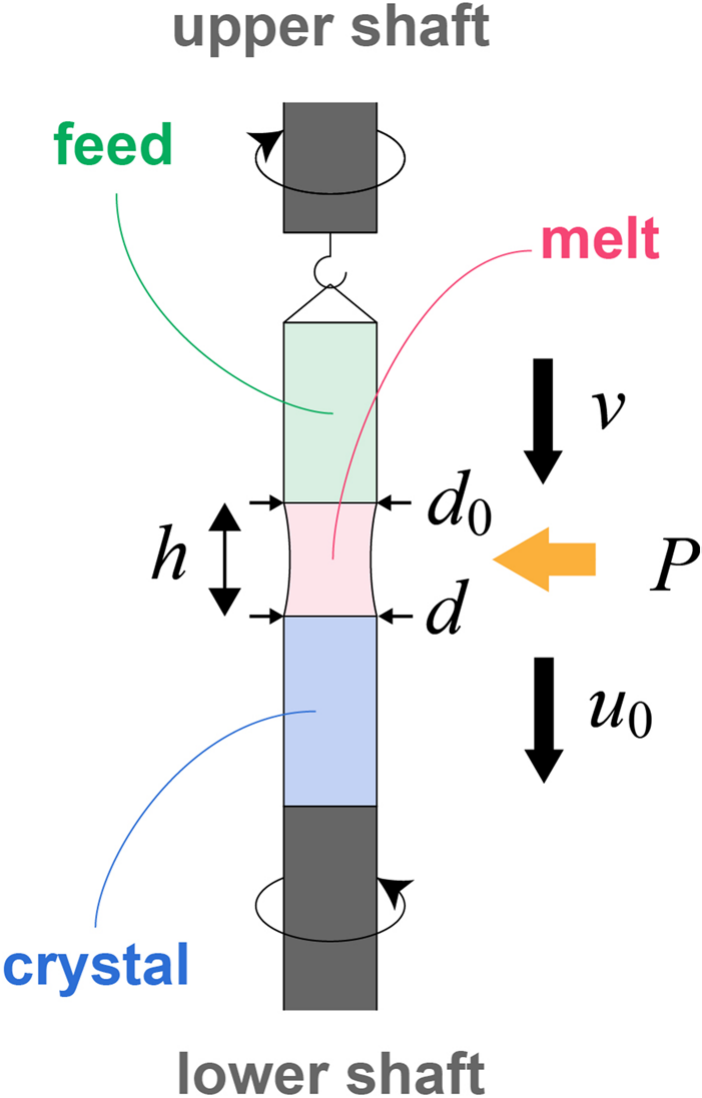
Schematic illustration of floating-zone crystal growth. A floating-zone melt with the height of *h* is formed by heater power *P*. A feed with diameter *d*_0_ and a crystal are moved downward with speeds *v* and *u*_0_, respectively. As a result, a crystal with diameter *d* is grown.

FZ crystal growth was developed to produce high-purity silicon single crystals without the molten zone touching with any foreign materials. Despite its advantage in growing high-purity crystals, enlargement of crystal diameter is difficult compared to other crystal growth technique such as Czochralski method. Relatively small silicon wafers are manufactured by FZ crystal growth using RF heating. Figure 1 shows a schematic illustration of FZ crystal growth. In this method, part of a polycrystalline rod is heated to create an FZ melt, and the upper (feed) rod and lower (seed) rod are moved downwards to maintain the FZ melt by surface tension; finally, the crystal grows on the seed rod. An operator controls the input parameters, such as the heating power and speed of the feed rod, so that the FZ melt does not separate or drip off. In addition, the operator must form a certain shape in which the crystal diameter is first reduced (called “necking”) and then increase the diameter of the crystal to obtain a single crystal. Since the dynamics of the melt state depending on the input parameters are non-linear and complicated, it is difficult to simulate the FZ crystal growth process, as has been achieved for other crystal growth methods^29–33^. Thus, it is necessary to predict the dynamics of FZ crystal growth from the operation trajectories. Due to the difficulty of acquiring numerous operation trajectories for FZ crystal growth, recently we proposed adaptation of the Gaussian mixture model (GMM) to predict the dynamics of FZ crystal growth, and demonstrated that GMM can precisely predict the operation trajectories from only five trajectories used for training^34^. In the present study, we constructed a control model by reinforcement learning using proximal policy optimization (PPO) and dynamics predicted by GMM.

### Reinforcement learning by PPO with GMM dynamics

For control of FZ crystal growth with a small number of demonstration trajectories, we applied reinforcement learning by PPO with the dynamics predicted by GMM. Here we describe how to construct a control model for FZ crystal growth combining GMM and PPO based on the literature^35^. The state of the floating-zone melt at time (*t* + 1), which is assumed to be composed of the height (*h*) and diameter of the grown crystal (*d*) and described as ***s***_***t*+1**_ = (*h*_*t*+1_, *d*_*t*+1_), is determined by the state of the melt at time *t* (***s***_***t***_), and input parameters, which include the power (*P*) and the movement speed of the feed (*v*), for example, and described as ***a***_***t***_ = (*P*_*t*_, *v*_*t*_).

1 $${\varvec{s}}_{{{\varvec{t}} + 1}} = {\varvec{f}}\left( {{\varvec{s}}_{{\varvec{t}}} , {\varvec{a}}_{{\varvec{t}}} } \right).$$

***f*** stands for the true dynamics for FZ crystal growth. Once the GMM is constructed from the demonstration trajectories, the state of the melt at time (*t* + 1) can be predicted by the state of the melt and the input parameters at time *t*:

2 $$\hat{\user2{s}}_{{{\varvec{t}} + 1}} = {\varvec{f}}_{{{\varvec{GMM}}}} \left( {{\varvec{s}}_{{\varvec{t}}} , {\varvec{a}}_{{\varvec{t}}} } \right).$$

The circumflex (^) represents that the value is predicted, and ${\varvec{f}}_{{{\varvec{GMM}}}}$ stands for a dynamics model trained by GMM. The details of the training of GMM are described in Ref. ^34^. In PPO, the parameterized policies function $\pi_{{{\varvec{\theta}}_{{\varvec{p}}} }} \left( {{\varvec{a}}_{{\varvec{t}}} {|}{\varvec{s}}_{{\varvec{t}}} } \right)$ with parameter vector ${\varvec{\theta}}_{{\varvec{p}}}$, which generates input values ***a***_***t***_ from the current state ***x***_***t***_ as a probability distribution, is iteratively optimized using a clipped surrogate objective $L^{CLIP} \left( {{\varvec{\theta}}_{{\varvec{p}}} } \right)$ instead of a policy gradient^35–37^.

3 $$L^{CLIP} \left( {{\varvec{\theta}}_{{\varvec{p}}} } \right) = {\hat{\mathbb{E}}}_{{{\varvec{s}}_{{\varvec{t}}} , {\varvec{a}}_{{\varvec{t}}} }} \left[ {\min \left( {r\left( {{\varvec{s}}_{{\varvec{t}}} ,{\varvec{a}}_{{\varvec{t}}} ,{\varvec{\theta}}_{{\varvec{p}}} } \right)\hat{A}\left( {{\varvec{s}}_{{\varvec{t}}} ,{\varvec{a}}_{{\varvec{t}}} } \right), {\text{clip}}\left( {r\left( {{\varvec{s}}_{{\varvec{t}}} ,{\varvec{a}}_{{\varvec{t}}} ,{\varvec{\theta}}_{{\varvec{p}}} } \right), 1 - \varepsilon, 1 + \varepsilon } \right)\hat{A}\left( {{\varvec{s}}_{{\varvec{t}}} ,{\varvec{a}}_{{\varvec{t}}} } \right)} \right)} \right]$$

4 $$r\left( {{\varvec{s}}_{{\varvec{t}}} ,{\varvec{a}}_{{\varvec{t}}} ,{\varvec{\theta}}_{{\varvec{p}}} } \right) = \frac{{\pi_{{{\varvec{\theta}}_{{\varvec{p}}} }} \left( {{\varvec{a}}_{{\varvec{t}}} {|}{\varvec{s}}_{{\varvec{t}}} } \right)}}{{\pi_{{{\varvec{\theta}}_{{\varvec{p}}} }}^{old} \left( {{\varvec{a}}_{{\varvec{t}}} {|}{\varvec{s}}_{{\varvec{t}}} } \right)}}.$$

$\in$ is a hyper-parameter determining a clipped region. $A\left( {{\varvec{s}}_{{\varvec{t}}} ,{\varvec{a}}_{{\varvec{t}}} } \right)$ is the advantage function described as follows:

5 $$A\left( {{\varvec{s}}_{{\varvec{t}}} ,{\varvec{a}}_{{\varvec{t}}} } \right) = Q\left( {{\varvec{s}}_{{\varvec{t}}} ,{\varvec{a}}_{{\varvec{t}}} } \right) - V\left( {{\varvec{s}}_{{\varvec{t}}} } \right),$$

where $Q\left( {{\varvec{s}}_{{\varvec{t}}} ,{\varvec{a}}_{{\varvec{t}}} } \right)$ is the state-action value function and $V\left( {{\varvec{s}}_{{\varvec{t}}} } \right)$ is the state-value function. Here we approximately represent $Q\left( {{\varvec{s}}_{{\varvec{t}}} ,{\varvec{a}}_{{\varvec{t}}} } \right)$ as follows:

6 $$Q\left( {{\varvec{s}}_{{\varvec{t}}} ,{\varvec{a}}_{{\varvec{t}}} } \right)\sim R_{t} \left( {{\varvec{s}}_{{\varvec{t}}} ,{\varvec{a}}_{{\varvec{t}}} } \right) + \gamma V\left( {{\varvec{f}}\left( {{\varvec{s}}_{{\varvec{t}}} ,{\varvec{a}}_{{\varvec{t}}} } \right)} \right),$$

where $R_{t} \left( {{\varvec{s}}_{{\varvec{t}}} ,{\varvec{a}}_{{\varvec{t}}} } \right)$ and γ are the reward function and the discount factor, respectively. The advantage function represents whether the action in which the input value ${\varvec{a}}_{{\varvec{t}}}$ is set under the melt state described as ${\varvec{s}}_{{\varvec{t}}}$ is preferable. When the action is preferable, the advantage function takes on a positive value and the policy is updated to increase the probability ratio $r_{t} \left( {{\varvec{\theta}}_{{\varvec{p}}} } \right)$ by maximizing the surrogate objective. On the other hand, the advantage function takes on a negative value and the policy is updated to decrease the probability ratio when the action is not preferable. Under conditions that the policy and dynamics are given, state sequences are generated as a probability distribution, and a state-value function can be calculated:

7 $$V_{\pi } \left( {{\varvec{s}}_{{\varvec{t}}} } \right) = {\mathbb{E}}_{{{\varvec{s}}_{{\varvec{t}}} }} \left[ {\mathop \sum \limits_{k = 0}^{T - t} \gamma^{k} R_{t + k} \left( {{\varvec{s}}_{{{\varvec{t}} + {\varvec{k}}}} ,\user2{ a}_{{{\varvec{t}} + {\varvec{k}}}} } \right)} \right].$$

where *T* is the length of the trajectories and the expected value is calculated over the probability distribution of the state sequences. In PPO, the state-value function is predicted from the training data without assigning a policy. Thus, the predicted state-value function parameterized with ${\varvec{\theta}}_{{\varvec{v}}}$ $\left( {\hat{V}_{{{\varvec{\theta}}_{{\varvec{v}}} }} \left( {{\varvec{s}}_{{\varvec{t}}} } \right)} \right)$ is optimized using the square-error loss $L^{VF} \left( {{\varvec{\theta}}_{{\varvec{v}}} } \right)$;

8 $$L^{VF} \left( {{\varvec{\theta}}_{{\varvec{v}}} } \right) = \left( {V_{\pi } \left( {{\varvec{s}}_{{\varvec{t}}} } \right) - \hat{V}_{{{\varvec{\theta}}_{{\varvec{v}}} }} \left( {{\varvec{s}}_{{\varvec{t}}} } \right)} \right)^{2} .$$

Once the state-value function is predicted, the action-value function $\left( {\hat{Q}\left( {{\varvec{s}}_{{\varvec{t}}} ,{\varvec{a}}_{{\varvec{t}}} } \right)} \right)$ and the advantage function $\left( {\hat{A}_{t} } \right)$ are also predicted by eqs. (6) and (5), respectively. In addition to the clipped surrogate objective and the state-value function error, an entropy bonus is added to ensure sufficient exploration and the following objective is maximized for each iteration in PPO^38^:

9 $$L\left( {{\varvec{\theta}}_{{\varvec{p}}} ,\user2{ \theta }_{{\varvec{v}}} } \right) = {\hat{\mathbb{E}}}_{{{\varvec{s}}_{{\varvec{t}}} , {\varvec{a}}_{{\varvec{t}}} }} \left[ {L^{CLIP} \left( {{\varvec{\theta}}_{{\varvec{p}}} } \right) - c_{1} L^{VF} \left( {{\varvec{\theta}}_{{\varvec{v}}} } \right) + c_{2} S\left[ {\pi_{{{\varvec{\theta}}_{{\varvec{p}}} }} } \right]\left( {{\varvec{s}}_{{\varvec{t}}} } \right)} \right],$$

10 $$S\left[ {\pi_{{{\varvec{\theta}}_{{\varvec{p}}} }} } \right]\left( {{\varvec{s}}_{{\varvec{t}}} } \right) = - \mathop \sum \limits_{{{\varvec{a}}_{{\varvec{t}}} }} \pi_{{{\varvec{\theta}}_{{\varvec{p}}} }} \left( {{\varvec{a}}_{{\varvec{t}}} {|}{\varvec{s}}_{{\varvec{t}}} } \right)\log \pi_{{{\varvec{\theta}}_{{\varvec{p}}} }} \left( {{\varvec{a}}_{{\varvec{t}}} {|}{\varvec{s}}_{{\varvec{t}}} } \right),$$

where *c*_1_ and *c*_2_ are weights. Maximizing $L^{CLIP} \left( {{\varvec{\theta}}_{{\varvec{p}}} } \right)$ means acquiring the optimized policy $\pi_{{{\varvec{\theta}}_{{\varvec{p}}} }} \left( {{\varvec{a}}_{{\varvec{t}}} {|}{\varvec{s}}_{{\varvec{t}}} } \right)$ as described in Eq. (3) and (4). Minimizing $L^{VF} \left( {{\varvec{\theta}}_{{\varvec{v}}} } \right)$ means that the state-value function is predicted without assuming a policy as described in Eq. (8). Maximizing $S\left[ {\pi_{{{\varvec{\theta}}_{{\varvec{p}}} }} } \right]\left( {{\varvec{s}}_{{\varvec{t}}} } \right)$ is an entropy of policy that is a regularization term for training. In PPO, ${\varvec{\theta}}_{{\varvec{p}}} ,\user2{ \theta }_{{\varvec{v}}}$ is simultaneously optimized in each iteration. Although *L*^*CLIP*^ depends on ${\varvec{\theta}}_{{\varvec{v}}}$ via $A\left( {{\varvec{s}}_{{\varvec{t}}} ,{\varvec{a}}_{{\varvec{t}}} } \right)$ and *L*^*VF*^ depends on ${\varvec{\theta}}_{{\varvec{p}}}$ via $V_{\pi } \left( {{\varvec{s}}_{{\varvec{t}}} } \right)$, in the iterative optimization process, ${\varvec{\theta}}_{{\varvec{v}}}$ in *L*^*CLIP*^ and ${\varvec{\theta}}_{{\varvec{p}}}$ in *L*^*VF*^ are regarded as constant values and not optimized, and the values of the previous step are applied.

In order to optimize the policy, it is necessary to specify the dynamics to calculate the state-value function by Eq. (7). In our algorithm, GMM dynamics were used for calculation of the state-value function. Thus, the algorithm is completely data-driven without any simulations, which is different from other methods such as the “sim-to-real” approach^39,40^. However, the GMM dynamics can reliably predict actual dynamics only in the vicinity of the training trajectories. Therefore, we proposed a method to optimize the policy near the training trajectories, where GMM dynamics reliably predict the actual dynamics, and obtain a policy that can transfer to actual FZ crystal growth. To search the policy space near the training trajectories, firstly, we performed pretraining to make the policy closer to the training trajectories. Secondly, we introduced the error from the averaged action sequences to the reward function in addition to the error from the ideal trajectory in the diameter $\left( {d_{t}^{ideal} } \right)$. The reward function used in our proposed algorithm is as follows:

11 $$R_{t} \left( {{\varvec{s}}_{{\varvec{t}}} ,{\varvec{a}}_{{\varvec{t}}} } \right) = - \left| {d_{t} - d_{t}^{ideal} } \right|^{2} - \lambda \left| {{\varvec{a}}_{{\varvec{t}}} - \overline{{{\varvec{a}}_{{\varvec{t}}}^{\user2{*}} }} } \right|^{2} .$$

$\overline{{{\varvec{a}}_{{\varvec{t}}}^{\user2{*}} }}$ and $\lambda$ denote the averaged action sequences of training trajectories and a weight.
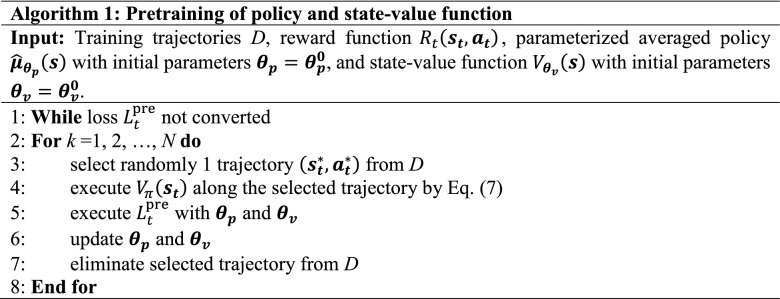


### Preparation of datasets

**Figure 2:**
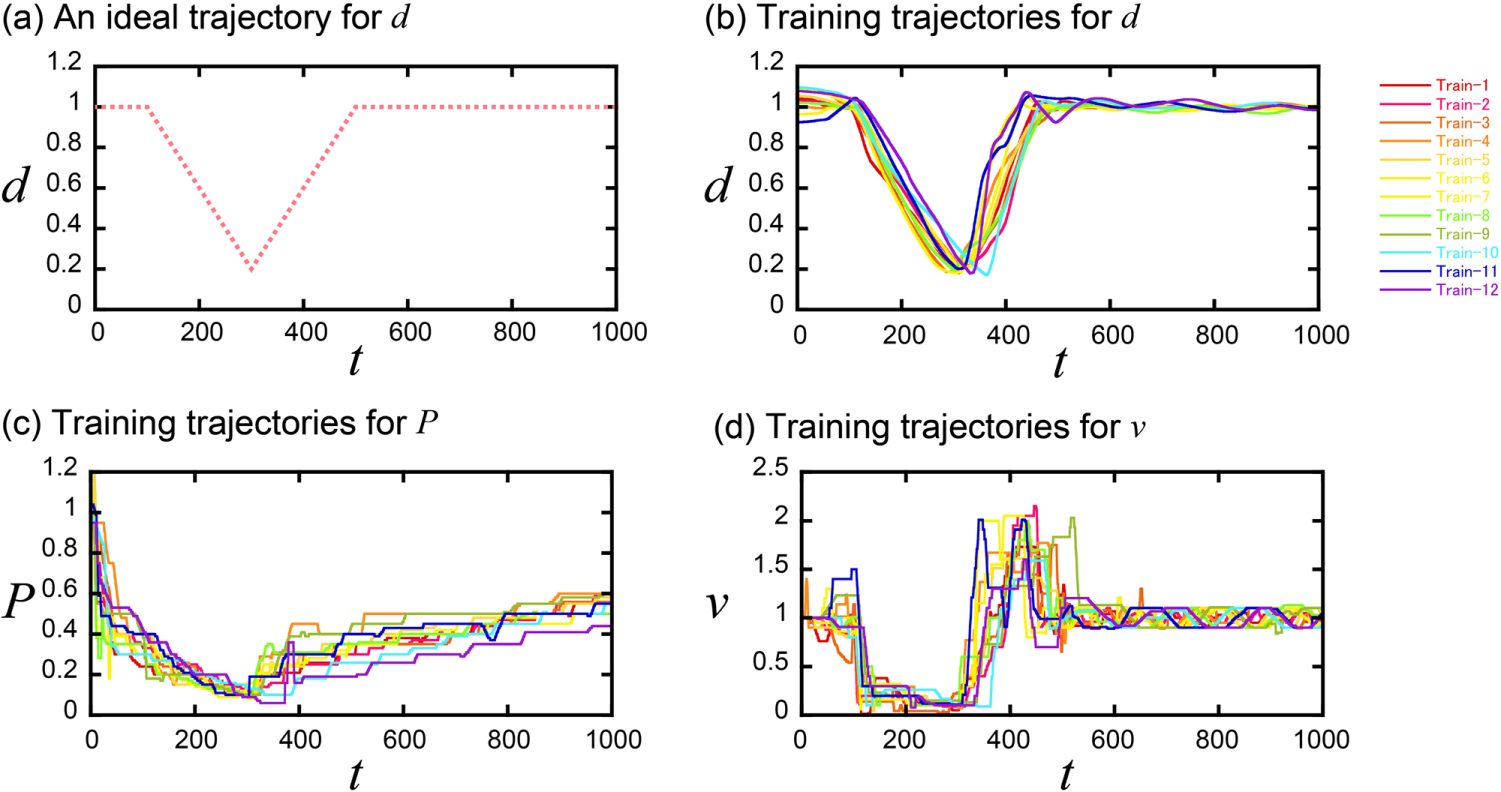
(**a**) An ideal trajectory for the diameter of the crystal, (**b**) trajectories of the diameter for training, and (**c**, **d**) operation trajectories of the power and movement speed of the feed.

To validate the automated control of FZ crystal growth by the algorithm using PPO with GMM dynamics, we prepared datasets for training ($D = \left\{ {\left( {{\varvec{s}}_{{\varvec{t}}}^{\user2{*}} ,{\varvec{a}}_{{\varvec{t}}}^{\user2{*}} } \right)_{1} ,\left( {{\varvec{s}}_{{\varvec{t}}}^{\user2{*}} ,{\varvec{a}}_{{\varvec{t}}}^{\user2{*}} } \right)_{2} , \ldots ,\left( {{\varvec{s}}_{{\varvec{t}}}^{\user2{*}} ,{\varvec{a}}_{{\varvec{t}}}^{\user2{*}} } \right)_{N} } \right\}$, where *N* is the number of training datasets) by use of an emulator program for FZ crystal growth with a given set of dynamics^34^. We prepared 12 datasets aiming to create an ideal crystal shape $\left( {d_{t}^{ideal} } \right)$ as shown in Fig. 2a considering the necking process for single crystal growth. Figure 2b–d show the prepared datasets aiming to create the ideal shape. The trajectories were different from each other and did not perfectly follow the ideal shape, because they were manually prepared.

### Policy optimization

Prior to the reinforcement learning, we constructed a data-driven prediction model for FZ crystal growth by GMM as we previously reported^34^. The number of Gaussian mixtures, which is a hyper-parameter of GMM, was set to 50. Since the prediction of the dynamics by GMM is reliable only near the training trajectories, the accuracy of the prediction is significantly poorer when the trajectories deviate greatly from the ideal trajectory as discussed in “Results and discussion” section especially with showing Fig. 4 in detail. If we start to optimize with the random default policy, the state sequences generated by GMM will be far from the actual state sequences and fail to reach the ideal trajectory shown in Fig. 2a. Thus, we performed pretraining using the training trajectories before optimization of the policy by PPO. In the pretraining, the policy was trained to become closer to the averaged action sequences of the training trajectories. The following loss function is minimized in the pretraining:

12 $$L_{t}^{{{\text{pre}}}} \left( {{\varvec{\theta}}_{{\varvec{p}}} ,\user2{ \theta }_{{\varvec{v}}} } \right) = L_{t}^{{{\text{policy}}}} \left( {{\varvec{\theta}}_{{\varvec{p}}} } \right) + L_{t}^{VF} \left( {{\varvec{\theta}}_{{\varvec{v}}} } \right),$$

13 $$L_{t}^{{{\text{policy}}}} \left( {{\varvec{\theta}}_{{\varvec{p}}} } \right) = \frac{1}{{2\sigma^{2} }} \left| {\hat{\user2{\mu }}_{{{\varvec{\theta}}_{{\varvec{p}}} }} \left( {{\varvec{s}}_{{\varvec{t}}} } \right) - {\varvec{a}}_{{\varvec{t}}}^{\user2{*}} } \right|^{2} ,$$

14 $$L_{t}^{{{\text{value}}}} \left( {{\varvec{\theta}}_{{\varvec{v}}} } \right) = \left( {\hat{V}_{{{\varvec{\theta}}_{{\varvec{v}}} }} \left( {{\varvec{s}}_{{\varvec{t}}} } \right) - V_{\pi } \left( {{\varvec{s}}_{{\varvec{t}}} } \right)} \right)^{2} ,$$

where *σ* and $\hat{\user2{\mu }}_{{{\varvec{\theta}}_{{\varvec{p}}} }} \left( {{\varvec{s}}_{{\varvec{t}}} } \right)$ represent the variance parameter and the predicted averaged values of inputs values under the state ${\varvec{s}}_{{\varvec{t}}}^{\user2{*}}$ in a training trajectory. $\hat{\user2{\mu }}_{{{\varvec{\theta}}_{{\varvec{p}}} }} \left( {{\varvec{s}}_{{\varvec{t}}} } \right)$ and $\hat{V}_{{{\varvec{\theta}}_{{\varvec{v}}} }} \left( {{\varvec{s}}_{{\varvec{t}}} } \right)$ are modeled by neural networks. The number, node number, and activation function of the hidden layers are 2, 64, and hyperbolic tangent (tanh), respectively. A sigmoid function is used as the activation function of the output layer of the policy network, and the output layer of the networks of the state-value function has no activation function. Both networks share weight values, except for the output layers. Training of the neural networks was performed by the Adam method with a learning rate of 1 × 10^–5^ and a batch size of 128^41^. The probabilistic policy was generated by the $\hat{\user2{\mu }}_{{{\varvec{\theta}}_{{\varvec{p}}} }} \left( {{\varvec{s}}_{{\varvec{t}}} } \right)$ and variance parameters.

15 $$\pi_{{{\varvec{\theta}}_{{\varvec{p}}} }} \left( {{\varvec{a}}_{{\varvec{t}}} {|}{\varvec{s}}_{{\varvec{t}}} } \right) = Gauss\left( {{\varvec{a}}_{{\varvec{t}}} {|}\hat{\user2{\mu }}_{{{\varvec{\theta}}_{{\varvec{p}}} }} \left( {{\varvec{s}}_{{\varvec{t}}} } \right),\sigma {\varvec{I}}} \right)$$

The detailed algorithm for pretraining the policy and state-value function is shown in Algorithm 1. After the pretraining of the policy, the policy was optimized by PPO while maximizing the objective shown in Eq. (8). Hyper-parameters used for the pretraining and training by PPO are summarized in Table 1. Our program about PPO for the FZ crystal growth trajectory is uploaded in GitHub^42^.

**Table 1:** Hyper-parameters used for the pretraining and training by PPO.

Parameter	Description	Value
*ϵ*	Parameter for a clipped region in the clipped surrogate objective	0.2
*γ*	Discount factor	0.05
*c*_1_	Weight parameters for the losses of PPO	0.2
*c*_2_		0.05
*λ*	Weight parameter for reward function	1
*σ*	Variance parameter for a probabilistic policy	0.05

## Results and discussion

**Figure 3:**
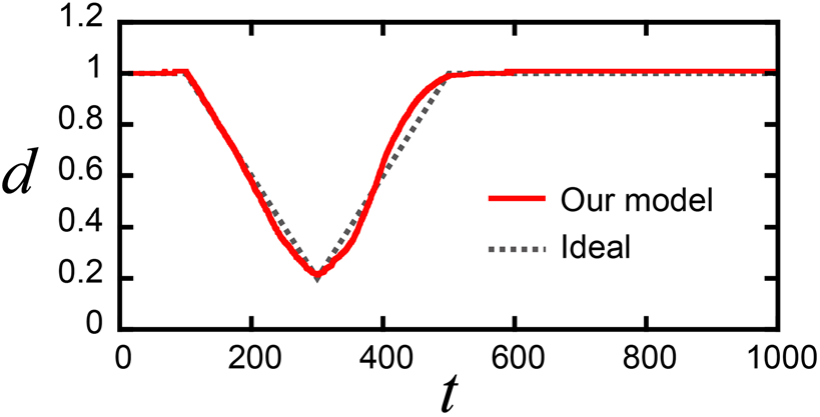
Trajectory of the diameter generated by the control model trained by our proposed algorithm.

Figure 3 shows the results of automated control by the trained policy with our proposed algorithm. Note that the training of the policy was performed by the dynamics predicted by GMM from only the training trajectories. The obtained trajectory follows the ideal trajectory well in terms of diameter. Table 2 summarizes the mean square error (MSE) from the ideal trajectory in diameter *d* for control by PPO and by humans (training trajectories). The deviation from the ideal trajectory for control by PPO is smaller than that for human control. We successfully constructed a control algorithm for FZ crystal growth with a defined ideal shape from several training trajectories.

**Table 2:** Mean square errors from the ideal trajectory.

	MSE (× 10^–3^)
Proposed	0.9
Manual	5.2

**Figure 4:**
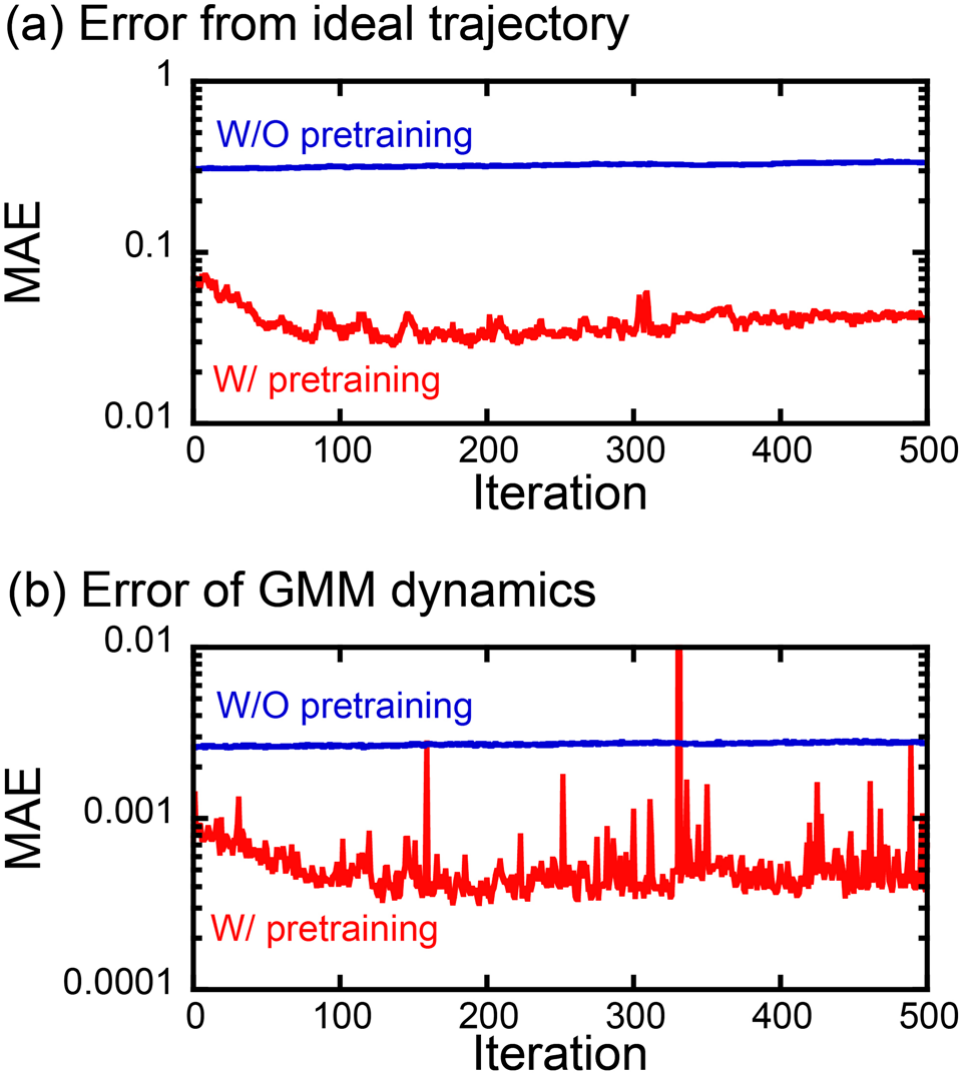
(**a**) Mean absolute error (MAE) from the ideal trajectory and (**b**) MAE of GMM dynamics along the generated trajectory during training with and without pretraining.

Pretraining of the policy before PPO is crucially important. Without pretraining, the learning of policy never progresses at all. Figure 4 shows the evolution of the averaged absolute error from the ideal trajectory in diameter *d* during training starting after pretraining and with randomly set initial values. With pretraining, the policy was well trained and the error decreased with increasing iteration and became saturated. On the other hand, the error from the ideal trajectory never decreased with increasing iteration without pretraining. Furthermore, the error of GMM dynamics from the true dynamics along the generated trajectory was consistently higher without pretraining than that after pretraining. These results indicate that the policy space was appropriately searched with GMM dynamics with high accuracy after the pretraining.

**Figure 5:**
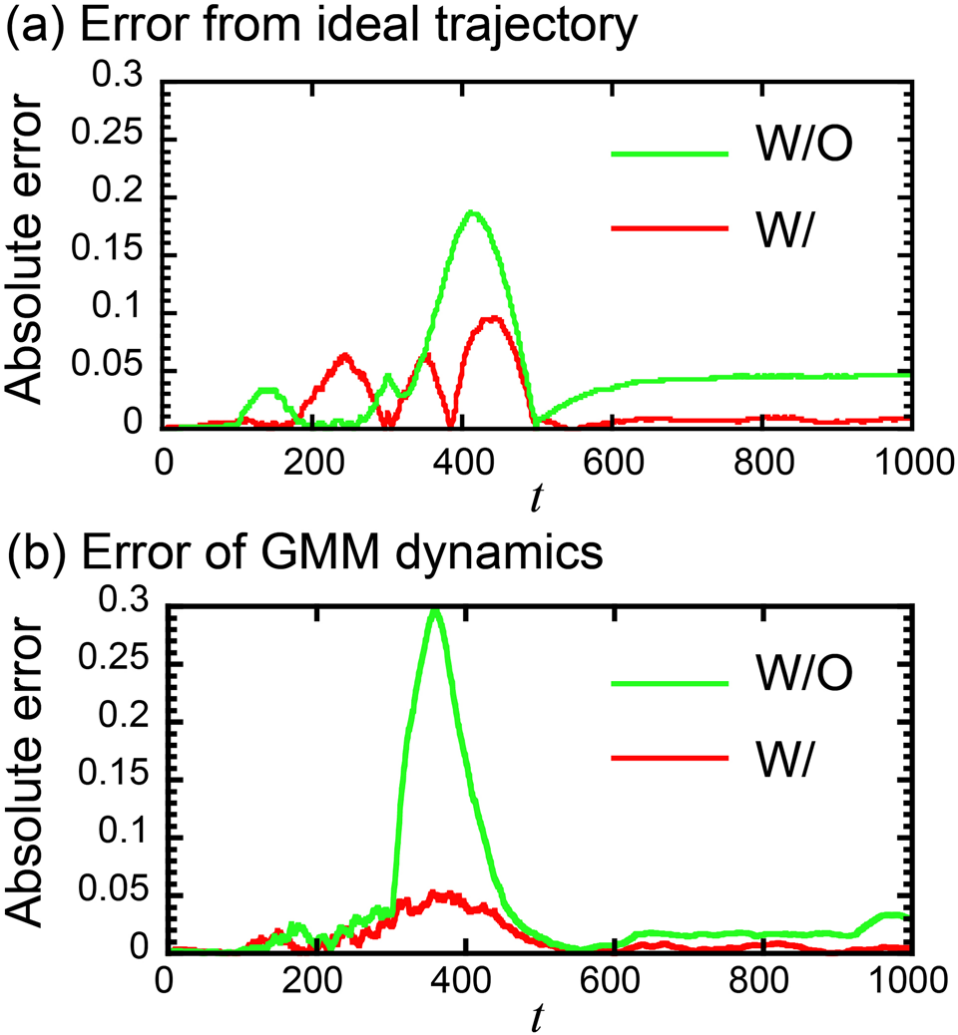
(**a**) Absolute errors from the ideal trajectory and (**b**) absolute errors of GMM dynamics along the trajectory generated with and without the second term in Eq. (11) in the reward function.

Design of the reward function, adding the error from the averaged action sequences in addition to the error from the ideal trajectory, is also important for policy optimization. Without the second term in Eq. (11), the deviation from the ideal trajectory is larger than our proposed reward shown in Eq. (11), especially around *t* = 400 and *t* > 600 (Fig. 5a). In these periods, the error of GMM dynamics for the trajectory generated by the reward without the second term in Eq. (11) is higher than that for the trajectory generated by our reward function (Fig. 5b). These results indicate that adding the second term in Eq. (11) successfully achieves optimization of the policy with the GMM dynamics within high accuracy by proper setting of the reward function.

The current demonstration shows that automated control of FZ crystal growth is possible by our proposed method from a small number of demonstration trajectories. Since our methods determine the policy based on the dynamics predicted by GMM, it is necessary to make the generated trajectory closer to the demonstration trajectory during policy optimization. Pretraining of the policy and proper design of the reward function successfully achieve optimization of the policy by the GMM dynamics within reliable prediction margins. Our proposed method will be able to be applied to other materials processes that require adaptive control according to the process status. Although the present demonstration was based on data obtained by an emulator program, our proposed methodology will work with actual FZ crystal growth.

## Conclusion

We have constructed a control model for FZ crystal growth by reinforcement learning using PPO with dynamics predicted by GMM. Our proposed method is completely data-driven and can construct the control model from only a small number of demonstration trajectories. We have verified our method to by a virtual experiment using the emulator program of FZ crystal growth. As a result, the control model was revealed to operate more accurately to follow an ideal trajectory in melt diameter than demonstration trajectories created by human operation. Since our methods determine the policy based on the dynamics predicted by GMM, it is necessary to make the generated trajectory closer to the demonstration trajectory during policy optimization. Pretraining of the policy near training trajectories and proper design of the reward function successfully achieved optimization of the policy by GMM dynamics within reliable prediction margins. Our proposed method will lead to the automation of materials processing in which adaptive operation is required and help realize high productivity in materials manufacturing. It is expected that the actual FZ crystal growth process can be automated from small number of demonstration trajectories operated by human.

## Data Availability

The data that support the findings of this study are available from the corresponding author, SH, upon reasonable request.
